# Bio-inspired electron-delivering system for reductive activation of dioxygen at metal centres towards artificial flavoenzymes

**DOI:** 10.1038/ncomms9509

**Published:** 2015-09-30

**Authors:** Yoann Roux, Rémy Ricoux, Frédéric Avenier, Jean-Pierre Mahy

**Affiliations:** 1 Laboratoire de Chimie Bioorganique et Bioinorganique, Institut de Chimie Moléculaire et des Matériaux d'Orsay (UMR 8182), Univ Paris Sud, Université Paris Saclay, rue du doyen Georges Poitou, 91405 Orsay, France

## Abstract

Development of artificial systems, capable of delivering electrons to metal-based catalysts for the reductive activation of dioxygen, has been proven very difficult for decades, constituting a major scientific lock for the elaboration of environmentally friendly oxidation processes. Here we demonstrate that the incorporation of a flavin mononucleotide (FMN) in a water-soluble polymer, bearing a locally hydrophobic microenvironment, allows the efficient reduction of the FMN by NADH. This supramolecular entity is then capable of catalysing a very fast single-electron reduction of manganese(III) porphyrin by splitting the electron pair issued from NADH. This is fully reminiscent of the activity of natural reductases such as the cytochrome P450 reductases with kinetic parameters, which are three orders of magnitude faster compared with other artificial systems. Finally, we show as a proof of concept that the reduced manganese porphyrin activates dioxygen and catalyses the oxidation of organic substrates in water.

## Abstract (web summary)

Activated atmospheric dioxygen is potentially an environmentally friendly oxidant. Here, the authors incorporate a flavin mononucleotide into a water soluble polymer, demonstrating single electron reduction of a metalloporphyrin centre, and subsequent activation of dioxygen for the oxidation of organic substrates.

## Graphical abstract

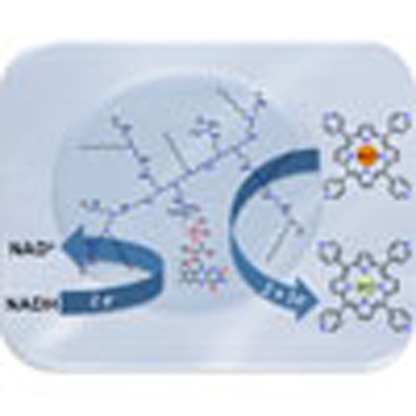

Selective catalytic oxidations are crucial reactions for the chemical industry, and new environment-friendly processes are highly needed to meet new standards for a sustainable growth. Yet, Nature has figured out an elegant manner to perform such reactions by the reductive activation of dioxygen at metal centres of multicomponent monooxygenases^1^,^2^. For example, in liver cells, cytochromes (P450) catalyse the oxidation of various xenobiotics and metabolites by the reductive activation of dioxygen at their haem cofactors, via the formation of high-valent iron-oxo intermediates, capable of transferring an oxygen atom into the C–H bond of substrates^2^. In bacteria, another protein complex, methane monooxygenase, catalyses the selective oxidation of methane into methanol via the reductive activation of dioxygen and the formation of a di-iron(IV) di-oxo intermediate^1^. Other metallic monooxygenases also activate dioxygen to perform oxidation reactions, and in all cases the two electrons needed at each catalytic cycle are provided to the metal centres by reductase proteins capable of harvesting electrons from NAD(P)H owing to their flavin cofactors FMN or FAD^3^,^4^. These cofactors collect electron pairs as hydride ions from NAD(P)H and ensure a stepwise electron transfer towards the catalytic metal centres of monooxygenases. The protein environment surrounding these flavin cofactors in the reductase proteins plays two key roles in the hydride transfer. It provides a binding site for NAD(P)H and it finely tunes the redox potential of flavin cofactors by creating a local specific microenvironment^5^,^6^.

These selective electron transfers constitute the main scientific lock in the perspective of performing a bio-inspired oxidation catalysis using dioxygen with metal-based catalysts. It has, indeed, been proven very difficult to provide electrons for the reductive activation of dioxygen, without quenching the active species formed on reaction with dioxygen. Hence, most of the bio-inspired catalysts developed so far had to shunt this reduction process by the use of hydrogen peroxide (see Fig. 1), and only a handful examples of catalysts have been described that are capable of using dioxygen directly^7^.

Nolte and co-workers have incorporated hydrophobic manganese porphyrins into vesicles in which electrons were provided by the oxidation of either hydrogen with colloidal Platinum^8^ or formate at rhodium complexes^9^,^10^. Other systems also used manganese porphyrins in combination with either nicotinamide and benzoic anhydride^11^, or pyruvate oxidase and flavin cofactors^12^. More recently, reductase proteins were substituted by ruthenium-based photosentisizers capable of gathering electrons on light irradiation and transferring them to cytochrome (P450) enzymes^13^,^14^,^15^. A similar strategy was also successful in the reduction of di-iron(III) complexes as functional mimics of methane monooxygenase^16^. However, despite the few examples cited above, efficient water-soluble systems capable of selectively driving electrons to metallic centres for the reductive activation of dioxygen are still highly needed.

In this perspective, multibranched polyethyleneimine (PEI), which are water-soluble polymers, can easily be modified with various chemical groups and they generate supramolecular entities bearing a specific local microenvironment, reminiscent to the active site of enzymes. These so-called ‘synzymes' (synthetic enzymes) have been shown to mimic various biological activities in the presence, or in the absence, of natural or handmade cofactors^17^,^18^,^19^,^20^,^21^,^22^,^23^,^24^. In terms of oxidation catalysis, the incorporation of polyoxometalate allowed, for example, the epoxidation of styrene in good yield in the presence of hydrogen peroxide^25^.

Herein, we describe the preparation and study of an artificial reductase on the basis of modified polyethyleneimine derivatized with guanidinium and octyl groups to bind the phosphate group of the FMN and bring this cofactor into a locally hydrophobic microenvironment. Once incorporated into the polymer, the FMN is able to efficiently collect electron pairs from NADH and insure a stepwise single-electron delivery to manganese(III) porphyrins with no use of other heavy metal, enzyme or additional chemical. Finally, we demonstrate that the obtained Mn(II) porphyrin can then activate dioxygen from the air and perform catalytic oxidation in water at room temperature.

## Results

### Construction of the artificial flavoenzyme

**Figure 2:**
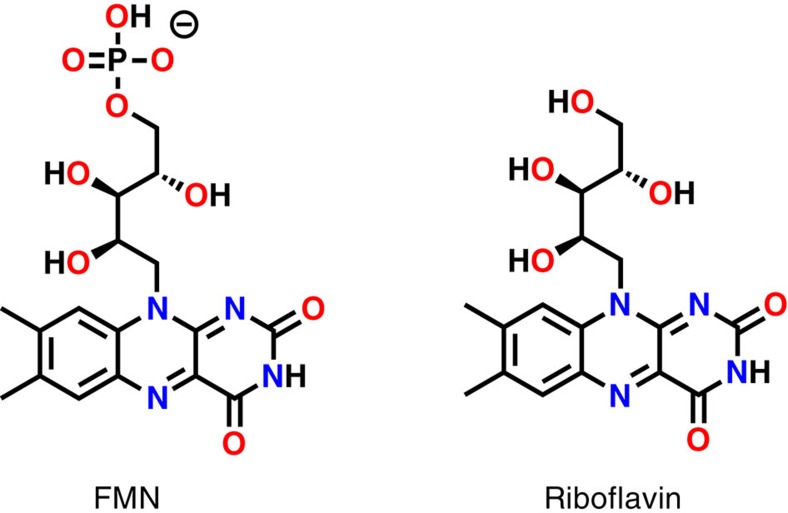
Flavin cofactors. FMN and riboflavin.

**Figure 3:**
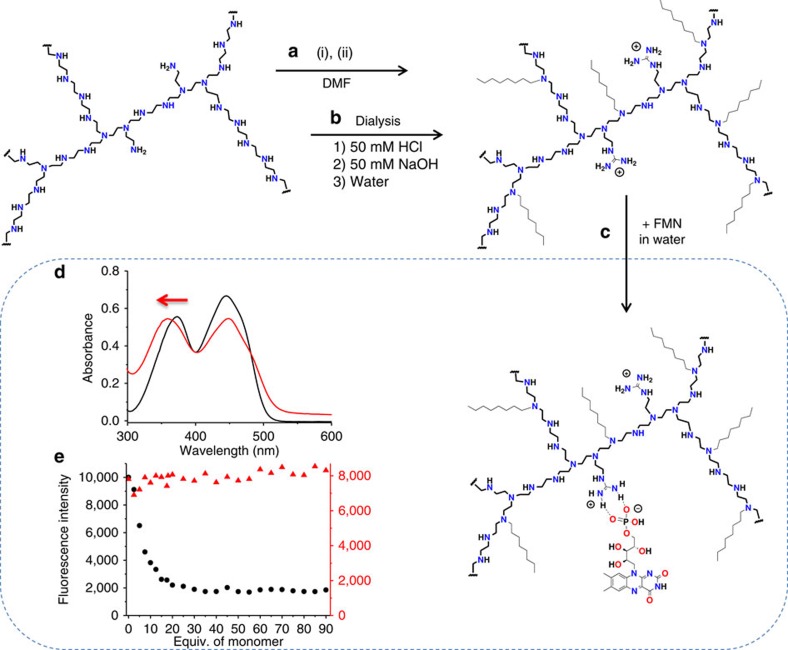
Preparation of the artificial flavoenzyme. (**a**) Synthesis of the modified PEI by reaction of a commercial 25-kDa multibranched PEI with (i) praxadine (1*H*-pyrazole-1-carboxamidine hydrochloride) and (ii) iodooctane in DMF at room temperature, (**b**) purification by extensive dialysis in 20% EtOH in 50 mM HCl; 50 mM HCl; water; 50 mM NaOH and water, (**c**) incorporation of FMN into the polymer, (**d**) ultraviolet-visible spectra of a 50-μM solution FMN in water (black trace) and in the presence of the modified PEI (4 mM in monomer; red trace). (**e**) Fluorescence intensity of 1 μM solutions of FMN (black circles) and riboflavin (red triangles) at 530 nm on excitation at 450 nm in the presence of an increasing amount of the modified PEI in water.

Since the flavin mononucleotide (FMN) cofactor (Fig. 2) usually binds to proteins by simple electrostatic interactions^26^, we decided to design bio-inspired artificial systems based on the same concept. To do so, a modified PEI bearing guanidinium and octyl groups was prepared as previously described, by reaction of the commercial PEI (25 kDa, multibranched) with praxadine and iodooctane in DMF^23^,^24^. Its purification was then performed by extensive dialysis in aqueous solutions (Fig. 3a,b). In this system, guanidinium groups were chosen for their specific affinity towards phosphate groups and their stability over a large pH range, whereas octyl groups were added for the creation of a locally hydrophobic microenvironment.

Flavin cofactors, such as FMN and riboflavin (Fig. 2), absorb light with two specific bands at 370 and 470 nm in aqueous solution. The former band at 370 nm is known to be sensitive to its environment, with a hypsochromic shift of ∼10 nm when solubilized in hydrophobic environments^27^,^28^. Figure 3d shows the ultraviolet-visible absorption spectra of FMN in pure water and in the presence of the modified PEI (guanidinium groups 0.2 equiv. per monomer/octyl groups 0.4 equiv. per monomer). As one can observe, the addition of the modified PEI to the aqueous solution of FMN induces a 12-nm shift of the band at 370 nm. When the same experiment was realized with riboflavin, that bears no phosphate group in its structure, no shift could be observed (Supplementary Fig. 1). This clearly indicates that the FMN is brought into an hydrophobic environment on the addition of the polymer (Fig. 3c), suggesting that its phosphate group interacts with the guanidinium groups of the polymer, whereas the riboflavin remains in solution. To confirm this incorporation, fluorescence spectra of both FMN and riboflavin were measured on the addition of increasing amount of the modified polymer. Figure 3e (and Supplementary Fig. 2) shows that only the fluorescence of the FMN is quenched by the presence of the polymer, indicating that riboflavin remains in aqueous solution, whereas the FMN interacts closely enough with the polymer to have its fluorescence quenched by its direct environment^29^.

### Reactivity of the artificial flavoenzyme with NADH

**Figure 4:**
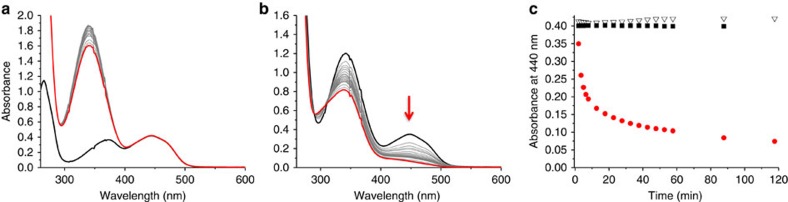
Influence of the modified PEI on the reduction of FMN by NADH. (**a**) Ultraviolet-visible spectra of a 50-μM deoxygenated aqueous solution of FMN before (black trace) and after addition of NADH (500 μM) followed for 2 h with the final curve in red. (**b**) Ultraviolet-visible spectra of a deoxygenated aqueous solution of FMN (50 μM) incorporated in the modified PEI (1.25 mM in monomer) in the presence of NADH (200 μM; (black traces) followed for 2 h with the final curve in red. (**c**) Time courses for the reduction of FMN (50 μM) by NADH (500 μM) in deoxygenated water only (reverse triangle), by NADH (200 μM) in presence of the modified polymer (1.25 mM in monomer; red circle), without NADH but in the presence of the modified polymer (1.25 mM; black square).

With the FMN buried into the locally hydrophobic microenvironment of the modified PEI, we endeavoured to study its reactivity with NADH in water. As one could expect, when a 200-μM (or even a 500 μM) water solution of NADH was added to a 50-μM FMN water solution, the hydride transfer between the two molecules did not occur, and no reduction of the FMN was observed (Fig. 4a). Conversely, when the FMN (50 μM) was incorporated into the modified PEI, the addition of NADH (200 μM) to the solution induced the decrease in the absorbance at 470 nm, indicating a reduction of the FMN (Fig. 4b)^30^. This reduction process was optimal for the addition of 4 equiv. of NADH with respect to the FMN, and did not improve for higher concentrations.

The initial rate for the NADH oxidation by the FMN was then measured by following the disappearance of NADH at 340 nm in the presence and in the absence of the modified PEI. The values obtained are, respectively, 4.00 (±0.10) × 10^−6^ and 2.00 (±0.20) × 10^−8 ^mol l^−1 ^min^−1^, giving a rate enhancement of 200 when the FMN was buried into the polymer. To compare this polymeric system to other NADH/FMN oxidoreductases, we also calculated the second-order rate constant of the reaction *k*=500 M^−1 ^s^−1^, which is, respectively, only three or four orders of magnitude slower than the natural NADH-specific FMN oxidoreductases from *Beneckea harveyi*^31^ and from *Pseudomonas putida*^32^, but is sevenfold faster compared with the semi-synthetic enzyme, flavopapain developed by Kaiser and co-workers^33^,^34^.

### Mechanistic implications on the hydride transfer

**Figure 5:**
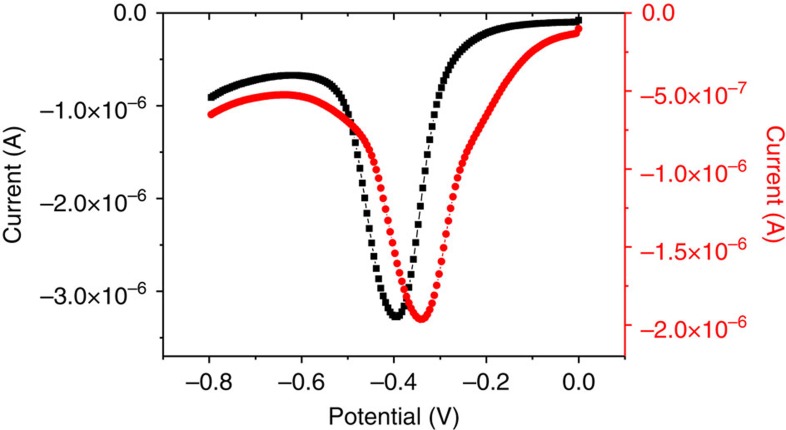
Electrochemistry. Square wave voltamogram of FMN (0.1 mM) in HEPES buffer (10 mM; pH=7) with KCl 10 mM (black squares) and square wave voltamogram of FMN (0.1 mM) in HEPES buffer (10 mM; pH=7) in the presence of the modified PEI (2,5 mM in monomer; red circles). Step potential 0.005 V, amplitude −0.020 V, frequency 25 Hz.

Since the FMN is not reduced by NADH in solution, one may suggest two effects of the modified PEI for this reduction. First, the polymer may favour the interaction between the two molecules into its locally hydrophobic microenvironment, by interaction of its guanidinium groups with the phosphate groups of the FMN and NADH. Alternatively, the incorporation of the FMN into the microenvironment of the polymer may change its redox potential and thermodynamically allow its reduction by NADH. To answer this question, we performed electrochemical analysis with the FMN free in solution, and with the FMN incorporated into the polymer (Fig. 5). The reduction potential measured by square wave voltammetry experiments for the FMN in aqueous solution was −395 mV versus Ag/AgCl (−203 mV versus Normal Hydrogen Electrode (NHE)), whereas in the presence of polymer the reduction potential was shifted to −330 mV versus Ag/AgCl (−138 mV versus NHE). This shift of 65 mV clearly indicates that the hydrophobic environment, created by the modified PEI, has a rather large influence on the reduction potential of the FMN. For comparison, the same experiment realized with the riboflavin shows only a 10-mV shift, which correlates with its none or poor incorporation into the polymer (Supplementary Fig. 3).

The thermodynamic redox potential for the NAD^+^/NADH couple was previously estimated based on equilibrium measurements for some enzyme-catalysed reactions to be −315 mV versus NHE^35^. This potential is thermodynamically low enough to reduce both the FMN in solution and the FMN buried into the polymer, implying that the 65-mV shift observed is not responsible for the generation of the reductase activity within the polymer. One can then conclude that the main role of the polymer is to bring together the two protagonists of the reaction in the same local hydrophobic microenvironment, and to facilitate the hydride transfer by lowering the energy barrier of the transition state. This is supported by results obtained with supramolecular flavin/NADH models, which clearly demonstrate the importance of an optimal relative disposition of the redox partners for the hydride transfer^36^. Finally, it is also worth to note that once reduced by the addition of NADH, the FMN was easily (re-)oxidized on exposure to dioxygen and then reduced again by the addition of NADH, making this system capable of multiple turn over reduction catalysis.

### Reduction of a water-soluble manganese porphyrin

**Figure 6:**
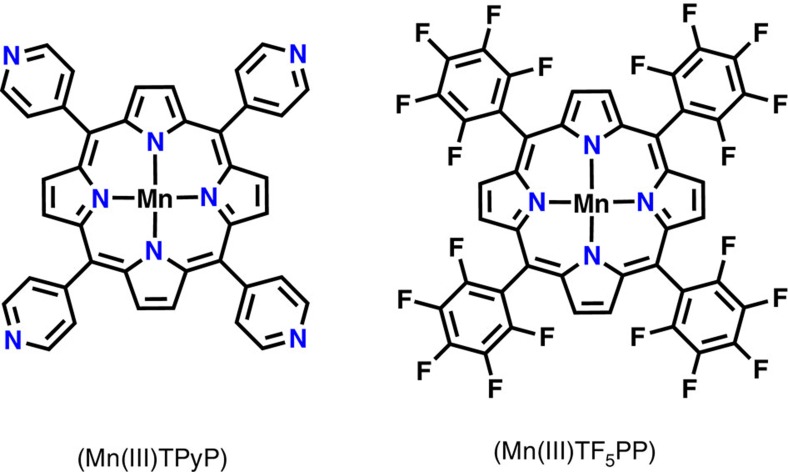
Manganese porphyrins used in this work. Manganese(III)-*meso*-tetrapyridinyl-porphyrin (Mn(III)TPyP) and manganese(III)-*meso*-tetra(pentafluorophenyl)porphyrin (Mn(III)TF_5_PP).

**Figure 7:**
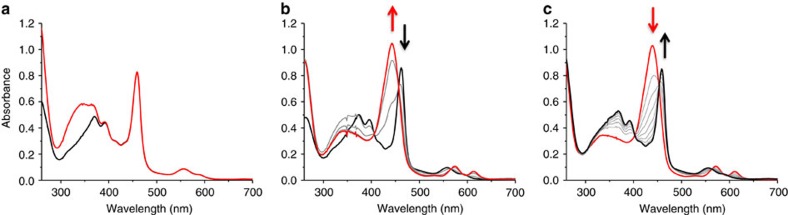
Reduction of a water-soluble manganese porphyrins. (**a**) Ultraviolet-visible spectrum of a deoxygenated aqueous solution of Mn(III)TPyP (10 μM) and FMN (10 μM; black trace) and its evolution for 1 h after addition of NADH (4 equiv.) at 20 °C (red trace). (**b**) Ultraviolet-visible spectrum of a deoxygenated aqueous solution of Mn(III)TPyP (10 μM) and FMN (10 μM) in the presence of the modified polymer (250 μM in monomer; black trace) and its evolution for 2 min after the addition of NADH (4 equiv.) at 20 °C (red trace). (**c**) Ultraviolet-visible spectra of a deoxygenated 10-μM aqueous solution of the reduced Mn(II)TPyP (10 μM) with FMN (10 μM; red trace) and its evolution for 6 min on exposure to O_2_ at 20 °C (black traces).

Since this artificial enzyme (modified PEI+FMN) was demonstrated to efficiently gather electrons from NADH, we also attended to study the possible transfer of those electrons towards a redox partner, such as a manganese(III)-*meso*-tetrapyridiniumylporphyrin (Mn(III)TPyP; Fig. 6). In this case, manganese porphyrins were chosen for the following two reasons: (1) Mn(III) and Mn(II) porphyrins have distinctive absorption bands at 470 and 440 nm, respectively, and (2) their absorption molar coefficients are high enough to be observed even in the presence of FMN. In a typical experiment, a stoichiometric amount of Mn(III)TPyP was added to a 10-μM aqueous solution of FMN before adding 4 equiv. of NADH, and the reaction was followed by monitoring the ultraviolet-Vis spectrum of the resulting solution as a function of time (Fig. 7). When the experiment was realized in the absence of the modified polymer, the characteristic band of the Mn(III) porphyrin at 470 nm did not change on addition of NADH, as well as for the following 60 min (Fig. 7a). Conversely, when the same experiment was realized in the presence of the modified polymer, the absorption band at 470 nm was rapidly replaced by a new band at 440 nm, characteristic of the reduced Mn(II)TPyP (Fig. 7b)^37^. The appearance of clear isosbestic points at 405, 460 and 570 nm during this fast process also demonstrated the direct one electron reduction of the manganese(III) porphyrin, without the observation of any other intermediate (Fig. 7b). Finally, once exposed to dioxygen, this Mn(II)TPyP was rapidly re-oxidized to Mn(III)TPyP with the same isosbestic points previously observed during the reduction process (Fig. 7c). It is worth noting that this one electron reduction of the manganese(III) porphyrin implies the splitting of the initial electron pair collected from NADH as an hydride ion.

**Figure 8:**
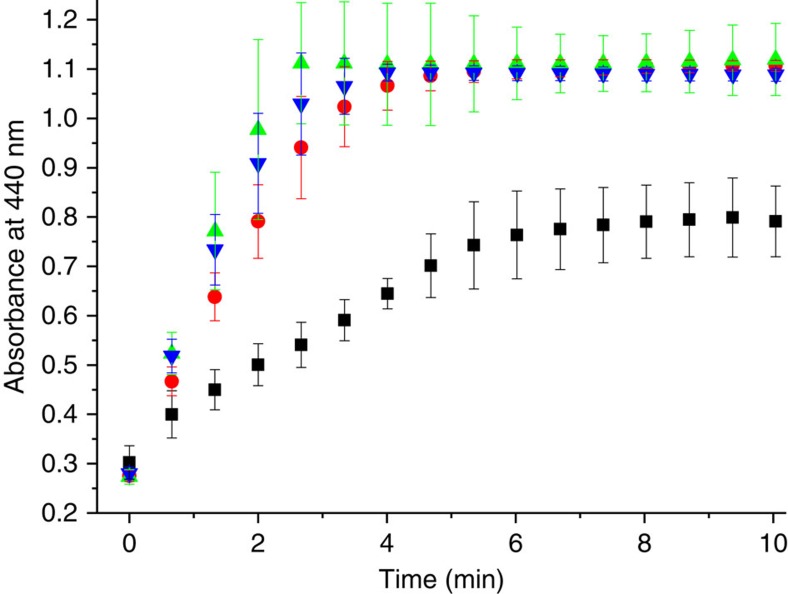
Titration of NADH for the reduction of a water-soluble manganese porphyrin. Time courses for the reduction of Mn(III)TPyP (10 μM) in the presence FMN (10 μM) and modified PEI (250 μM in monomer) by following the absorbance of Mn(II)TPyP at 440 nm on the addition of 0.25 equiv. (black square), 0.5 equiv. (red circle), 1 equiv. (green up triangle) and 2 equiv. (blue down triangle) of NADH in deoxygenated water at 25 °C (all experiments were realized in triplicate).

Splitting electron pairs from NADH, to perform single-electron transfer, is one of the key features involved in important biological processes. Hence, we decided to titrate the reduction process of the Mn(III)TPyP by successive addition of NADH in the presence of the modified polymer to better understand this electron transfer. Figure 8 shows the time course for this reduction by following the formation of the Mn(II)TPyP at 440 nm. As one can observe, below 0.5 equiv. of NADH added, the reduction of the Mn porphyrin was not completed. However, the addition of 0.5 equiv. of NADH was enough to fully reduce the Mn porphyrin, while the addition of larger amount of the reducing agent only changed the kinetics of the reduction. This titration demonstrates that one electron pair, issued from one NADH molecule, is enough to reduce two Mn porphyrin molecules via the FMN cofactor, implying the splitting of the electron pair during the process. This is fully reminiscent of the biological activity of cytochrome P450 reductases and will be of great interest in the perspective of the development of new bio-inspired reduction systems, especially for the development of environment-friendly oxidation catalysts.

**Figure 9:**
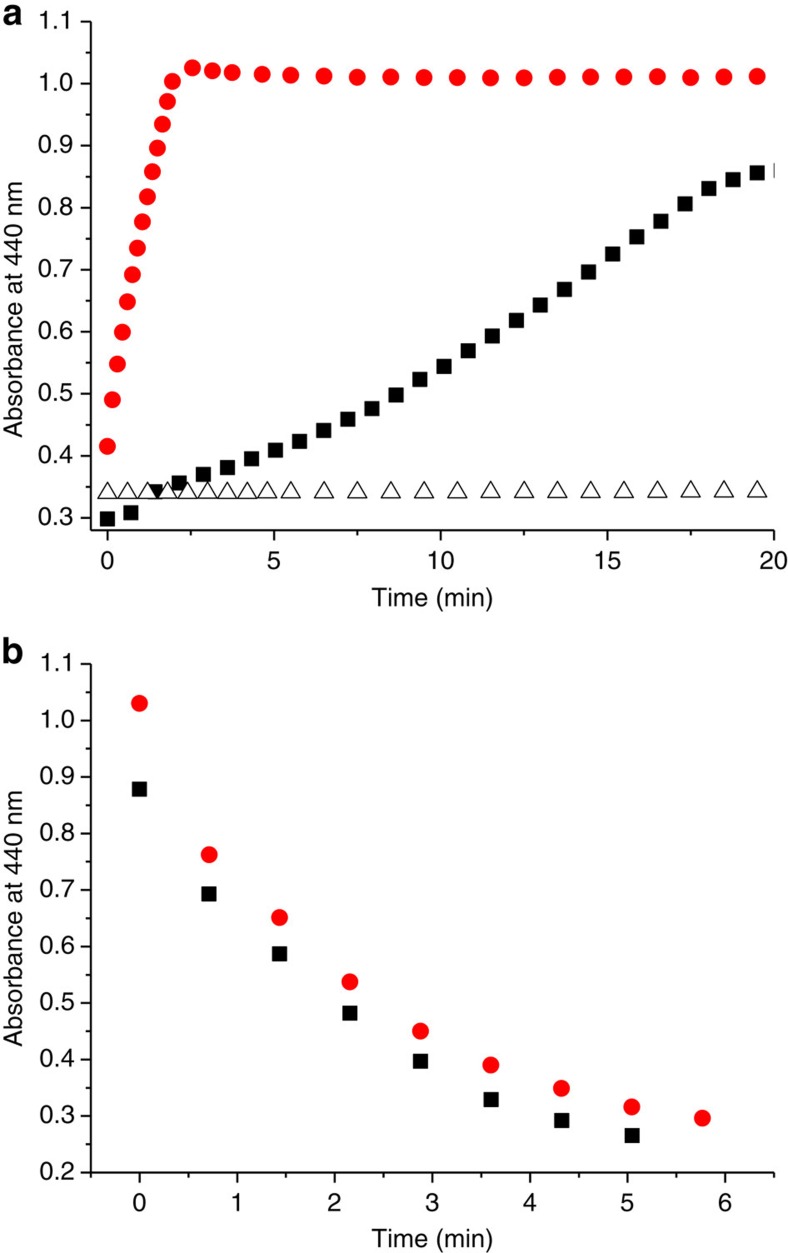
Reduction and O_2_ re-oxidation of a water-soluble manganese porphyrin. (**a**) Time course of the Mn(III)TPyP (10 μM) reduction on addition of NADH (4 equiv.) under inert conditions by following the formation of the reduced Mn(II)TPyP at 440 nm, in the presence of the modified PEI (250 μM) and FMN (10 μM; red circle), in the presence of the commercial PEI (250 μM) and FMN (10 μM; black square) and in the presence of FMN (10 μM) only (open triangle). (**b**) Time course of the Mn(II)TPyP (10 μM) oxidation on exposure to dioxygen in the presence of the modified PEI (250 μM; red circle) and in the presence of the commercial PEI (250 μM; black square) by following its absorbance at 440 nm.

In terms of kinetics, Fig. 9 shows that the reduction of the Mn(III) porphyrin, in the presence of the modified polymer, is completed within 2 min after the addition of NADH (4 equiv.), whereas almost no reduction is observed in pure water. The initial rates of the reactions were measured and gave 3.90 (±0.60) × 10^−6 ^M min^−1^ in the presence of the modified polymer and 9.65 (±0.57) × 10^−10 ^M min^−1^ in water only. In these conditions, second-order rate constant calculated from the equation *v*=*k*[MnTPyP][NADH] gave *k*_2_=163 M^−1 ^s^−1^ and *k*_2_=0.04 M^−1 ^s^−1^, which correspond to a rate enhancement of 4 × 10^3^. If the commercial polymer is used instead of the ‘synzyme', the reaction does occur, but much more slowly compared with the modified polymer (Fig. 9a). This is in good agreement with the fact that the commercial polymer is positively charged in water, which allows its interaction with the FMN and NADH, favouring the hydride transfer. However, the lack of specificity for the interaction, combined with the absence of hydrophobic microenvironment, drastically slows down the reduction process. Therefore, the screening of polymers derivatized with various amount of octyl groups will be, in the future, a precious tool to better understand the effect of a locally hydrophobic microenvironment on the kinetics of electron transfer in water. Finally, it is worth to note that, on exposure to dioxygen, the oxidation of the Mn(II) porphyrin took place at the same rate, whether the experiment was performed with the modified or with the commercial polymer (Fig. 9b). This suggests that the oxidation kinetic mostly relies on the diffusion of dioxygen in water, and that the Mn porphyrin is not incorporated within the polymer but equally dispersed in solution in both cases.

Examples of artificial flavoreductase capable of reducing metal complexes are very scarce, and only two systems are provided with kinetic information about the reduction process. A synthetic multicomponent redox system, bearing a manganese porphyrin and an amphiphilic flavin, both inserted within a phospholipidic bilayer, was described in association with a pyruvate oxidase in charge of delivering electrons^12^. In this case, the appearance of the manganese(II) porphyrin was followed at 440 nm and was shown to take more than 3 h to be completed. Similarly, a flavocyclodextrin system was demonstrated to be able of reducing a manganese(III) porphyrin using synthetic nicotinamide with second-order rate constants sixfold better compared with the FMN in solution^38^. Since nicotinamide molecules used in those studies are not always the same, the best way to compare the activity of our polymer with the literature is to compare the values of the rate enhancement of the various systems with one of FMN in solution. In that case, the ‘synzyme' appears to be three orders of magnitude better compared with the flavocyclodextrin^38^.

### Dioxygen activation in water

**Figure 10:**
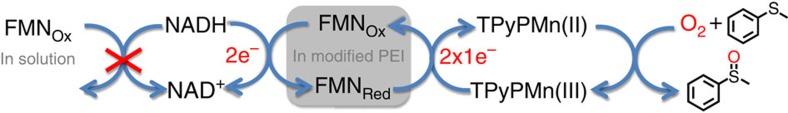
Schematic representation of the complete catalytic system. The artificial flavoenzyme collects electrons from NADH and provides a single-electron flow to the Mn(III) porphyrins to activate dioxygen and perform catalytic oxidation in water.

Finally, since the Mn(III)TPyP is efficiently reduced by the ‘synzyme' in the presence of NADH and then (re)oxidized by dioxygen, we endeavoured to perform catalysis in water by simple use of dioxygen as the oxidant, using thioanisole as the substrate. In a typical experiment, 1,000 equiv. of substrate (100 mM final) were added to a water solution of the ‘synzyme' (modified PEI+FMN) and Mn(III)TPyP (1 equiv. versus FMN; 0.1 mM final), before adding 10 equiv. of NADH (1 mM final). On addition of NADH, the reddish solution turned green after a few minutes, and the mixture was allowed to stir at room temperature for 5 h under oxygenated atmosphere (O_2_ at 1 atm). The mixture was then filtered through a short silica gel column to remove the polymer, the FMN and the Mn porphyrin, before being analysed using gas chromatography. Under these conditions, the oxidation of thioanisole gave the sulfoxide as an unique product of the reaction, but the yield was not significantly higher than the control experiment without the polymer. Other control experiments, performed either without Mn porphyrin, or without NADH, did not generate oxidation product at all. Since the use of Mn(III)TPyP was not adapted to catalysis, we also tested a fluorinated Mn porphyrin (manganese(III)-*meso*-tetra(pentafluorophenyl)porphyrin (Mn(III)TF_5_PP)), which is known as one of the best porphyrin for oxidation catalysis. In this case, the selectivity of the reaction did not change but the yield was improved to 28% (value obtained after subtracting the blank value obtained without the polymer). This rather hydrophobic porphyrin was initially solubilized in acetonitrile and added to the aqueous solution to obtain a 9:1 (water:MeCN) final mixture. It is worth noting that in the absence of the polymer, this Mn porphyrin precipitated a few minutes after its incorporation into the aqueous solution. Conversely, the solution was stable for hours in the presence of the polymer, demonstrating that the locally hydrophobic microenvironment of the polymer allows the solubilization of the Mn porphyrin in aqueous medium^39^. This modest yield of 28% for catalytic oxidation is not surprising since three major scientific locks have to be tackled simultaneously: (1) selective electron delivery at metal centres, (2) reductive activation of dioxygen at this metal centre and (3) catalysis in water. Nevertheless, these results clearly demonstrate the achievement, by a water-soluble handmade system, of a complete catalytic cycle reminiscent of the activities of iron monooxygenases such as cytochromes P450 (Fig. 10). This system is also simple enough to be easily adapted to various catalytic processes requiring a steady and accurate input of electrons.

## Discussion

In summary, we have described the incorporation, by electrostatic interactions, of FMN cofactors into a water-soluble PEI bearing guanidinium and octyl groups. This macromolecular entity was demonstrated to be capable of efficiently collecting electron pairs from NADH and rapidly delivering single electrons towards redox cofactors such as manganese(III) porphyrin, with a rate enhancement of 4 × 10^3^ compared with FMN in solution. This activity is fully reminiscent of cytochrome P450 reductase and opens new horizons in terms of green catalytic oxidation systems on the basis of the reductive activation of dioxygen at metal centres. As a proof of concept, this artificial reductase was associated with manganese(III) porphyrin in solution and was exposed to dioxygen in the presence of NADH. Under these conditions, the entire system was able to catalyse sulfoxidation reaction in water, at room temperature and using dioxygen as the sole source of oxygen. This system could easily be adapted to the use of other catalysts such as mononuclear or dinuclear iron complexes, but also copper complexes and other catalysts using electrons for their catalytic activity in water.

## Methods

### Instrumentation

All ultraviolet-visible measurements were performed with a Varian carry 300-bio ultraviolet-vis spectrophotometer in cuvettes equipped with septa for experiments under inert atmosphere. Fluorescence spectra were obtained with a Tecan infinite M200 pro-plate-reader. Gas chromatography analyses were performed using a SHIMADZU GC-2014A.

### Synthesis of the artificial flavoenzyme

FMN, riboflavin and NADH were purchased from commercial suppliers and used without further purification. The TPyP and the TF_5_PP were synthesized according to literature^40^,^41^, and the metal insertion was carried out according to the method in ref. 42.

The modified polymer (guanidinium groups 0.2 equiv. per monomer/octyl groups 0.4 equiv. per monomer) was synthesized from multibranched PEI (25 kDa) that was dissolved in DMF to give a final PEI concentration of 8.6 mg ml^−1^ (200 mM in monomer residues). For derivatization with guanidinium groups, 4 equiv. of triethylamine per monomer was added to the PEI solution, and a freshly prepared DMF solution of 1-H-pyrazole-1-carboxamidin hydrochloride (1 ml at 1.2 mol l^−1^) was added under vigorous stirring to 30 ml of the PEI solution. This reaction mixture was then left under stirring overnight at room temperature. For alkylation, 0.433 ml of iodooctane was added dropwise under vigorous stirring, which was continued for 4 days at room temperature. For purification, the crude reaction mixture was diluted (1:2) into hydrochloric acid (50 mM) and transferred to a dialysis tube (Spectra/Por membrane, Mw cutoff 14,000). The resulting solution was dialysed under slow stirring against each of the following buffers for at least 2 h: 20% EtOH in 50 mM HCl; 50 mM HCl; distilled water (twice); 50 mM NaOH (twice); and water (three times).

### Reduction of the artificial flavoenzyme

For the reduction of FMN by NADH (with or without polymer), solutions of FMN (2.5 mM), modified polymer (20 mM), NADH (12.5 mM) and water were prepared separately and degassed for 1 h. In parallel, a ultraviolet-visible cuvette (2.5 ml), equipped with a septum, was degassed and a solution of FMN (50 μM) and modified PEI (1.2 mM) was prepared before injection of NADH (200 μM) through the septum. The same experiment was realized without polymer.

### Electrochemistry

For electrochemistry experiments, a solution of FMN (0.1 mM) was prepared in HEPES buffer (10 mM). The square wave voltamogram was recorded under argon before and after the addition of modified polymer (2.5 mM) between 0 and −0.8 V. In absence of the polymer, the experiment was realized with KCl 10 mM as an electrolyte, which is not needed in the presence of the positively charged polymer. Measurements were realized with a carbon electrode, electrode of reference (Ag/AgCl) and counter electrode (Pt). (Step potential 0.005 V, amplitude 0.020 V, frequency 25 Hz.).

### Reduction of a water-soluble manganese porphyrin

For the reduction of a water-soluble manganese porphyrin, an aqueous solution of FMN (10 μM), modified polymer (250 μM in monomer) and MnTPyP (10 μM) was prepared directly in a quartz cuvette (2.5 ml) equipped with a septum. In parallel, a solution of NADH (2.5 mM) was prepared in water, and both solutions were degassed for 30 min. Then, the kinetic spectrum for the reduction of MnTPyP was recorded at 20 °C under argon after the addition of NADH (40 μM). The same experiment was realized without polymer. After reduction, the cuvette was opened to the air to follow the oxidation process.

### Dioxygen activation and catalysis

Catalysis experiments were realized in 1.5-ml vials in aqueous solutions of FMN (100 μM), MnTPyP (100 μM), modified polymer (2.5 mM in monomer) and thioanisole (100 mM) with a final volume of 1 ml. NADH (1 mM) was then added under vigorous stirring, and the mixture was left under stirring for 5 h at room temperature under O_2_ atmosphere (1 atm). For GC analysis, acetophenone was added to the solution as internal standard and the mixtures were filtered through a short silica gel column (on Pasteur pipette) before injection in a Zebron ZB Semi Volatiles column (30 m × 0.25 mm × 0.25 μm). GC conditions were as follows: 100–130 °C, 5 °C min^−1^, then 130–300 °C, 50 °C min^−1^, then hold for 3 min. Injector and FID temperature was 300 °C. Retention times (min) are as follows: acetophenone, 4.03; thioanisole, 4.32; thioanisole sulfoxide, 7.56; and thioanisole sulfone, 8.04.

## Additional information

**How to cite this article:** Roux, Y. *et al*. Bio-inspired electron-delivering system for reductive activation of dioxygen at metal centres towards artificial flavoenzymes. *Nat. Commun*. 6:8509 doi: 10.1038/ncomms9509 (2015).

## Supplementary Material

Supplementary Information Supplementary Figures 1-3

## Figures and Tables

**Figure 1:**
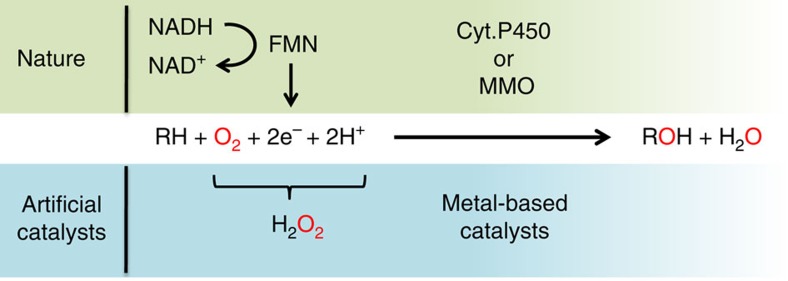
Natural and bio-inspired catalytic oxidations. Catalytic oxidation performed at the active site of monooxygenase enzymes by reductive activation of dioxygen, and the corresponding peroxide shunt used by most of the handmade catalysts developed so far.
