# Supplementary material for: Bio-inspired electron-delivering system for reductive activation of dioxygen at metal centres towards artificial flavoenzymes
Source: Nat Commun. 2015 Sep 30;6:8509. doi: 10.1038/ncomms9509 (PMC4598860; doi:10.1038/ncomms9509)
Supplement: Supplementary Information — Supplementary Figures 1-3 [file ncomms9509-s1.pdf]

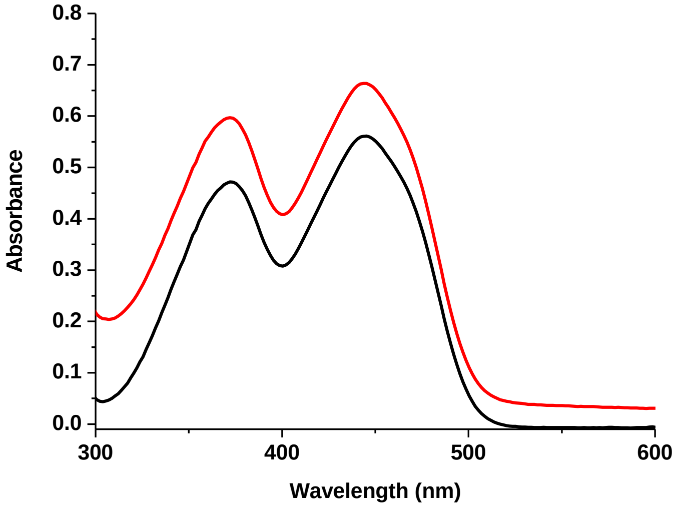

Supplementary Figure 1. UV-Visible spectra of a 50 μM solution of riboflavine in water (black trace) and in the presence of the modified PEI at a concentration of 4 mM in monomer (red trace).

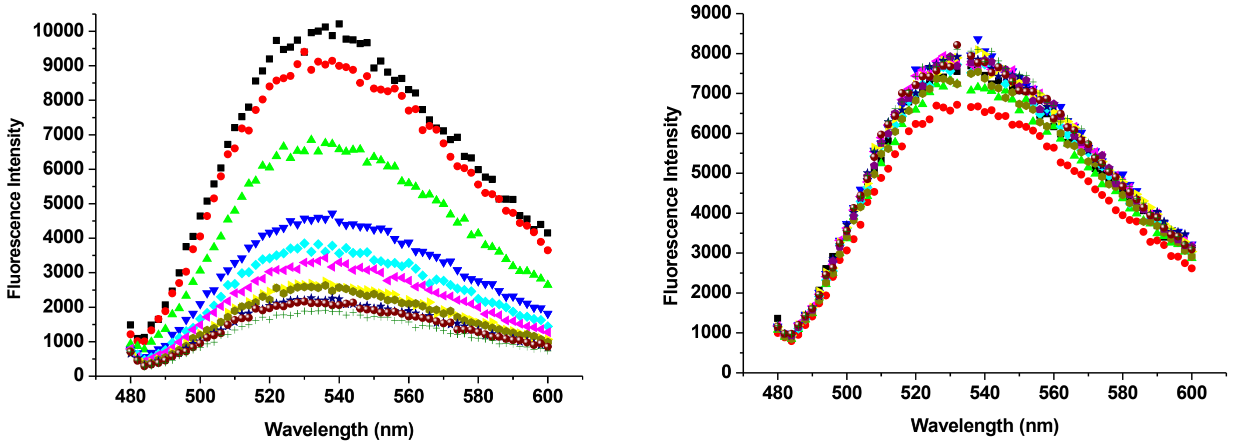

Supplementary Figure 2. Fluorescence spectra of 1 μM solutions of FMN (left) and of riboflavin (right) upon excitation at 450 nm in the presence of increasing amounts of the modified PEI (from 0 to 35 μM in monomer) in water. Quenching of fluorescence experiments were realised in a 96 well black plates at an excitation wavelength of 450 nm with 25 flashes per second, spell time of 2 ms, a time measure of 20 μs and a lag time of 8 μs. Maximum of emission was measured at 530 nm.

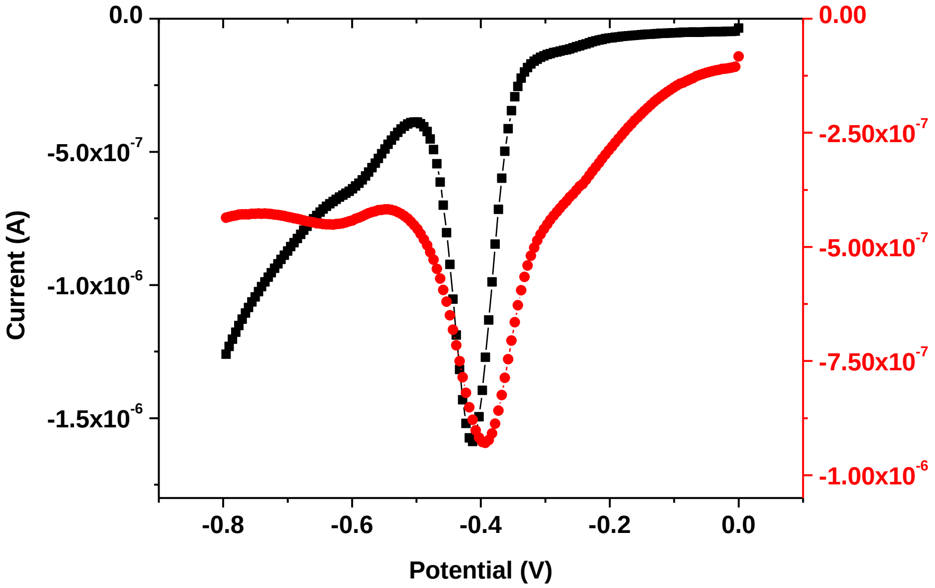

Supplementary Figure 3. Square wave voltamogram of riboflavin (0.1 mM) in HEPES buffer (10 mM; pH=7) with KCl 10 mM (black square) and square wave voltamogram of riboflavin (0.1 mM) and in HEPES buffer (10 mM; pH=7) in the presence of the modified PEI (2,5 mM in monomer) (red circle) Step potential 0.005V, amplitude -0.020V, frequency 25 Hz.
